# Homology modeling, molecular docking, and molecular dynamics simulations elucidated α-fetoprotein binding modes

**DOI:** 10.1186/1471-2105-14-S14-S6

**Published:** 2013-10-09

**Authors:** Jie Shen, Wenqian Zhang, Hong Fang, Roger Perkins, Weida Tong, Huixiao Hong

**Affiliations:** 1 Division of Bioinformatics and Biostatistics, National Center for Toxicological Research, U.S. Food and Drug Administration, 3900 NCTR Road, Jefferson, AR 72079, USA; 2 Office of Scientific Coordination, National Center for Toxicological Research, U.S. Food and Drug Administration, 3900 NCTR Road, Jefferson, AR 72079, USA

## Abstract

**Background:**

An important mechanism of endocrine activity is chemicals entering target cells via transport proteins and then interacting with hormone receptors such as the estrogen receptor (ER). α-Fetoprotein (AFP) is a major transport protein in rodent serum that can bind and sequester estrogens, thus preventing entry to the target cell and where they could otherwise induce ER-mediated endocrine activity. Recently, we reported rat AFP binding affinities for a large set of structurally diverse chemicals, including 53 binders and 72 non-binders. However, the lack of three-dimensional (3D) structures of rat AFP hinders further understanding of the structural dependence for binding. Therefore, a 3D structure of rat AFP was built using homology modeling in order to elucidate rat AFP-ligand binding modes through docking analyses and molecular dynamics (MD) simulations.

**Methods:**

Homology modeling was first applied to build a 3D structure of rat AFP. Molecular docking and Molecular Mechanics-Generalized Born Surface Area (MM-GBSA) scoring were then used to examine potential rat AFP ligand binding modes. MD simulations and free energy calculations were performed to refine models of binding modes.

**Results:**

A rat AFP tertiary structure was first obtained using homology modeling and MD simulations. The rat AFP-ligand binding modes of 13 structurally diverse, representative binders were calculated using molecular docking, (MM-GBSA) ranking and MD simulations. The key residues for rat AFP-ligand binding were postulated through analyzing the binding modes.

**Conclusion:**

The optimized 3D rat AFP structure and associated ligand binding modes shed light on rat AFP-ligand binding interactions that, in turn, provide a means to estimate binding affinity of unknown chemicals. Our results will assist in the evaluation of the endocrine disruption potential of chemicals.

## Background

The potential for environmental and exogenous chemicals to interfere with hormone (endocrine) systems in both humans and wildlife has been an international scientific debate that has persisted for many decades [1]. Concerns associated with so-called endocrine disruptors (EDs) led to requirements in the Food Quality Protection Act of 1996 (FQPA1996) and the Safe Drinking Water Act Amendments of 1996 (SDWA Amendments 1996) for the Environmental Protection Agency (EPA) to screen and identify substances with hormonal effects. In accordance to these acts, the EPA developed the Endocrine Disruptor Screening Program (EDSP) to identify chemicals with potential for endocrine disruption [2]. The endocrine system comprises glands that produce hormones and the receptors that respond to those hormones [3], as well as other proteins that can bind the hormones in serum [4]. EDs can mimic endogenous hormone ligands acting as agonists, partial agonists, or antagonists, altering gene expression and homeostasis, resulting in adverse developmental, reproductive, neurological and immune system effects [5]. The ability of chemicals to bind hormone receptors is a major mechanism for altering endocrine activity. Exogenous chemical binding to ER is particularly concerning due to potential for altering normal estrogen signaling through genomic and non-genomic pathways [6-8].

AFP is a serum protein in mammals that is produced in the yolk sac and liver of a developing fetus [9]. It is a member of the albuminoid gene superfamily. In human, AFP has long been used as a serum marker for fetal defects and tumor progression [10]. In rodents, AFP sequesters endogenous estrogen [11], blocking entry where it would otherwise induce ER-mediated responses.

To better estimate ER binding potential of a chemical in rodents, it is important to know its rat AFP binding properties. In 1972, Uriel *et al*. first reported the binding properties of rat AFP to steroidal chemicals using an immuno-autoradiographic assay [12]. Thereafter, rat AFP has been used to study *in vitro *binding and *in vivo *transport of steroids [13-15]. Recently, we developed a competitive binding assay using rat amniotic fluid, and used it to measure AFP binding affinities to 125 chemicals in 15 diverse, structural categories [4]. Some 53 chemicals from 13 categories bound AFP, while 72 chemicals did not. Importantly, we previously also measured ER binding affinity for 114 of the 125 chemicals, of which 47 bound both ER and AFP, 42 bound only ER, and 19 bound neither AFP nor ER. ER binding was not measured for the remaining 11 chemicals. These data provide a large dataset to study ligand to rat AFP binding preferences. Two possible estrogen-binding sites on rodent AFP have been proposed based on experimental binding data [16,17]; however, no 3D structures for rat AFP (with or without bound ligands) were available to confirm the binding sitesand structural dependencies on site activity.

Computational methods to predict protein structure and ligand-protein interaction have been successfully applied in biochemical research for decades. Terentiev et al. have reported the 3D homology model of human AFP in 2012[18]. In their work, the 3D model of human AFP was built to study the interaction of human AFP and diethylstilbestrol (DES), which is a strong ER binder and a validated endocrine disruptor. Herein, we built a 3D structure for rat AFP using homology modeling with subsequent optimization with MD simulations. Using the optimized 3D rat AFP structure, the rat AFP-ligand binding modes for 13structurally diverse rat AFP binders were calculated with molecular docking, followed by refinement using MD simulations.

This study reports the first 3D structure of rat AFP that was built through homology modeling and optimized using MD simulations. The 3D structure was demonstrated to be stable and trustworthy. Based on the 3D structure, the ligand binding modes of 13 structural diverse rat AFP binders were elucidated using molecular docking. Moreover, rat AFP conformation changes induced by ligands during the MD simulations were observed. Ligand binding free energies of the rat AFP binders were calculated using the MM-GBSA method and revealed that rat AFP can accommodate structurally diverse ligands having different electrostatic and hydrophobic properties. Glu206 was found to be the most important residue for rat AFP binding to flavones and mycoestrogens, while Tyr168 was most important for binding benzophenones and coumarin.

## Materials and methods

### Study design

**Figure 1:**
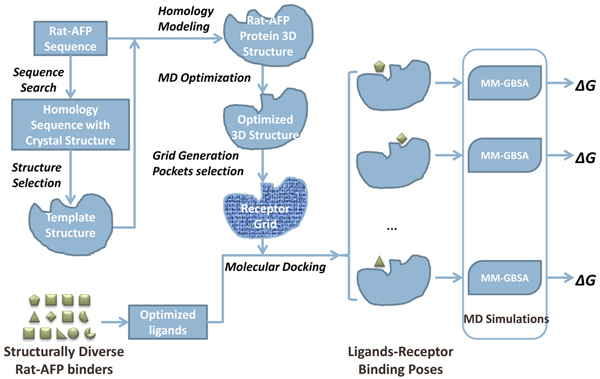
**Study design and overall workflow**. BLAST was used for the rat AFP protein sequence against PDB to select a template sequence for homology modeling. The initial 3D structure obtained from homology modeling was further optimized using MD simulation. The optimized 3D structure was used as the docking receptor. Thirteen structurally diverse rat-AFP binders were selected from our recently reported results [4] and were docked into the receptor. Finally, the ligand binding free energy for the complex was calculated based on the MD simulations.

The study design and workflow are depicted in Figure 1. Briefly, the rat AFP protein sequence was first searched in the protein data bank (PDB) using BLAST to select a template protein highly homologous to rat AFP. An initial 3D rat AFP structure was then built using homology modeling. The 3D structure was subsequently optimized with MD simulation. The optimized 3D rat AFP structure was used to define a binding grid for docking analyses. Thirteen structurally diverse rat AFP binders were selected from our previously reported results[4] and their 3D structures were built and optimized. The optimized 3D structures of the 13 rat AFP binders were then docked into the docking grid. The rat AFP-ligand complexes from docking analyses were further optimized through MD simulations. Finally, the rat AFP-ligand binding mode and the ligand binding free energy for each AFP binder were analyzed based on the trajectories of MD simulations.

### Homology modeling

The sequence of rat AFP was downloaded from the universal protein resource (Uniprot) [19] (entry: P02773). The template for sequence alignment was identified through searching rat AFP on PDB using the BLASTp [20] program provided by Uniprot with default parameters. The 3D structure of rabbit serum albumin (Uniprot ID: G1U9S2) was downloaded from PDB (PDB ID: 4V5F) [21] as the template structure. The homology model of rat AFP was built with Prime 3.1 in Schrödinger Suite (Schrödinger, LLC, New York, NY). The secondary structure of rat AFP was predicted using the SSpro program bundled with Prime. The target (rat AFP) and template (rabbit serum albumin) sequences were aligned using the ClustralW method employed in Prime, followed by manual adjustment to avoid big gaps in the secondary structure domain. The original ligand in the template structure was removed before homology modeling.

### MD optimization

The initial 3D structure of rat AFP obtained from homology modeling was optimized using MD simulation. The Amber ff03.R1 [22] force field was applied to the protein. Topology and parameter files were generated using the LEaP program[23]. MD simulation was conducted using Amber11 [23]. The 3D rat AFP structure was surrounded by a truncated octahedron periodic box of TIP3Pwater molecules with a margin of 10.0 Å along each dimension. Sodium ions were added to the system to maintain its charge neutrality. All covalent bonds to hydrogen atoms were constrained using the SHAKE algorithm[24]. Electrostatic interactions were calculated using the particle-mesh Ewald (PME) algorithm [25]with a cutoff of 10 Å for Lennard-Jones interactions. Periodic boundary conditions were applied to avoid edge effects. Prior to MD production, 500 steps of steepest-descent minimization and 1500 steps of conjugated gradient minimization were applied to the solvent and the entire model system, respectively. The entire system was heated from 0 to 300 K gradually over 30 ps (picoseconds) using the NVT (constant volume and normal temperature) ensemble with the solutes restrained by a weak harmonic potential. During the heating, time constant for heat bath coupling for the solute was set as 1.0.Afterward, 140 ps of equilibrations were carried out in the NPT (constant normal pressure and normal temperature) ensemble via three steps: first the solutes were restrained while the waters and counter-ions were equilibrated in the first 20 ps; then the side chains of rat AFP were relaxed in the next 20 ps; last, all the restraints were removed in the last 100 ps. Finally, 10 ns (nanoseconds) MD simulations were conducted at 1 atm and 300 K under the NPT ensemble with a time step of 2 fs (femtoseconds). The temperature was controlled using Langevin dynamics. The coordinates of all atoms in the system were saved every 1 ps during the entire MD simulations.

### Ligand preparation

**Figure 2:**
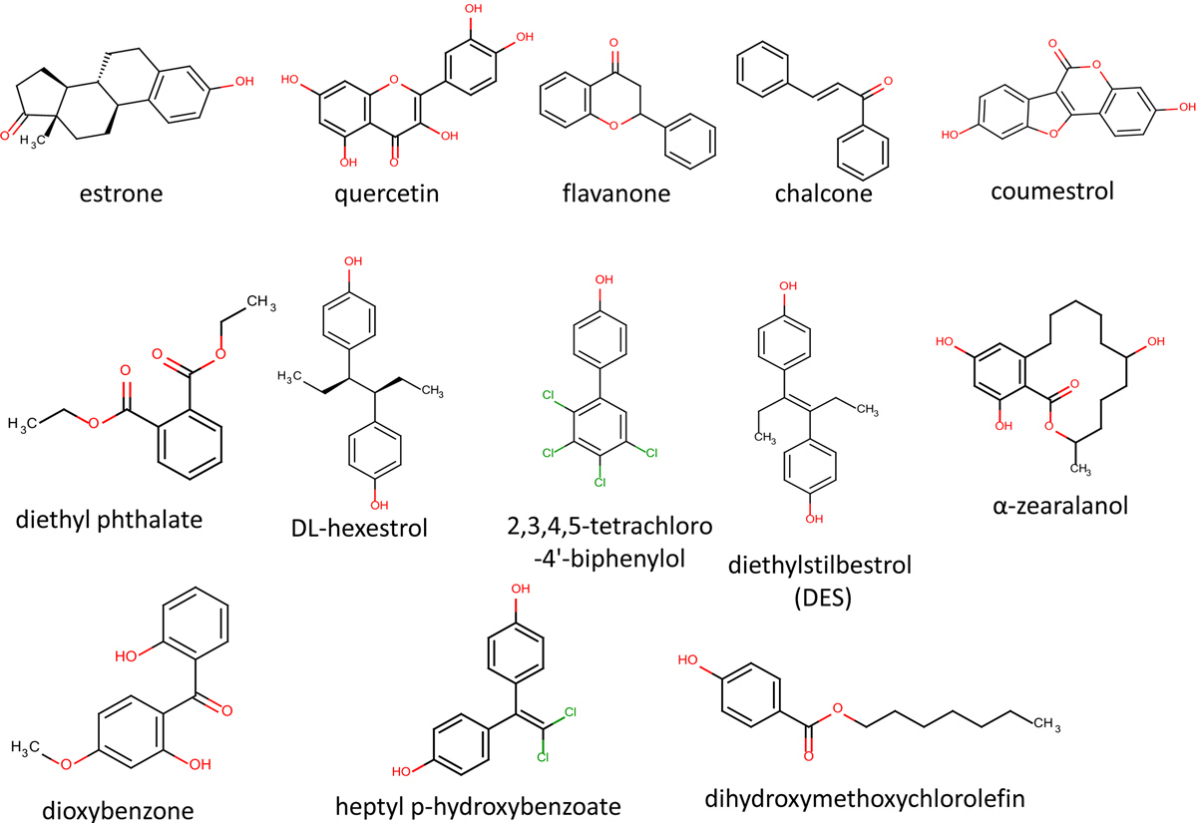
**Chemical structures and names of the 13 representative rat-AFP binders used in this study**.

Our previous study [4] identified 53 rat AFP binders from 13 structural categories. The most potent binder in each category was selected as the exemplars for docking analyses. The structures and chemical names are given in Figure 2. The 3D structures for these were built using Ligprep2.5 in Schrödinger Suite with an OPLS_2005 force field. Their ionization states were generated at pH7.0±2.0 using Epik2.2 in Schrödinger Suite. Up to 32 possible stereoisomers per ligand were retained.

### Docking grid generation

Prior to molecular docking, the optimized 3D rat AFP structure was prepared using the "Protein Preparation Wizard" workflow in Schrödinger Suite. Bond orders were assigned and hydrogen atoms were added to the protein. The structure was then minimized to reach the converged root mean square deviation (RMSD) of 0.30 Å with the OPLS_2005 force field. Probable ligand binding sites (on or near the protein surface) were searched using SiteMap2.6 in Schrödinger Suite. Then, contour maps of hydrophobic and hydrophilic fields were generated. The hydrophilic maps were further divided into donor, acceptor, and metal-binding regions. Finally, all the sites were assessed by calculating various properties. Thereafter, a docking grid was defined using "Receptor Grid Generation" in Schrödinger Suite. The grid enclosing box was centered in rat AFP with an internal size of 14 × 14 × 14 (x × y × z, Å). The grid was made large enough to cover all the potential ligand binding sites in the protein. Since the active site of rat AFP is not tight and encapsulated, the scaling factor of Van der Waals radius was set as 1.0 with a partial atomic charge less than 0.15 e, which means no scaling is done in this case.

### Molecular docking

The optimized 3D structures of the 13 rat AFP binders were docked into the docking grid in the 3D structure of rat AFP using Glide5.8 in Schrödinger Suite with standard precision (SP). The final binding poses with the top glide score were selected for further optimization through MD simulations.

### MD simulations

MD simulations were performed for the 13 rat AFP-ligand complexes using the similar protocol described in the MD optimization section. The ligands were optimized at the Hartree-Fock level with the 6-31G(d,p) basis set using Gaussian 09 (Gaussian, Inc., Wallingford, CT). Restrained electrostatic potential (RESP) charges were then calculated using the B3LYP/cc-pVTZ quantum mechanical method. The general Amber force field (GAFF) was applied to the complexes [26]. Topology and parameter files were generated using the LEaP program [24].The force field parameters of ligands wereobtained from the Antechamber modulein the AmberTools1.5 program [24]. The system minimization, heating, and equilibration were carried out in the same manner used for the optimization of 3D rat AFP structure described above. The MD simulations were performed for up to 60 ns for each complex system. The coordinates of atoms in the complex were saved every 10ps during the simulations.

### Binding free energy calculation

The binding free energy (Δ*G*_binding_, equation (1)) for each rat AFP-ligand complex system was calculated using the MM-GBSA approach [27]. A total of 1000 snapshots were taken in atime slice of 50-60 ns MD simulation trajectory to calculate the MM-GBSA free energy difference. For each snapshot, the rat AFP-ligand binding free energy was calculated using equation (1).

(1) $$\text{Δ}G_{\text{binding}}=G_{\text{complex}}-\left(G_{\text{protein}}+G_{\text{ligand}}\right)$$

where *G*_complex_, *G*_protein _and *G*_ligand _are free energies of complex, protein and ligand, respectively. Each of the components was estimated using equation (2),

(2) $$\text{Δ}G\;=\;\text{Δ}G_{gas}+\text{Δ}G_{solv}-T\text{Δ}S$$

where Δ*G*_gas _is the molecular mechanics free energy in gas phase, including electrostatic and van der Waals contributions (equation (3)). Δ*G*_solv _is the solvation free energy, including polar (Δ*G*_GB_) and nonpolar (Δ*G*_SA_) contributions (equation (4)).

(3) $$\text{Δ}G_{\text{gas}}=\text{Δ}E_{\text{electrostatic}}+\text{Δ}E_{\text{vdw}}$$

(4) $$\text{Δ}G_{\text{solv}}=\text{Δ}G_{\text{GB}}+\text{Δ}G_{\text{SA}}$$

(5) $$\text{Δ}G_{\text{SA}}=\gamma \times \text{SASA}+b$$

The polar contribution (Δ*G*_GB_) was calculated with the modified GB model described by Onufriev *et al. *[28] using *ε*_w_= 80 and *ε*_p_=1.0. SASA is the solvent-accessible area that is determined using the linear combination of pairwise overlaps method [29]. The surface tension proportionality constant γ and the free energy of nonpolar solvation for a point solute *b *were set to 0.0072 kcal/mol/Å^2 ^and 0.00 kcal/mol, respectively. The radius of the probe sphere used to calculate SASA was set to 1.4 Å. The entropy calculation is only a rough estimation with normal mode analysis. The calculated free energies were used for comparisons among the 13 rat AFP binders. Therefore, the entropy term was not included in our analyses. The final binding free energy for a rat AFP binder was the average value from the 1000 snapshots in the last 10 ns of MD simulations.

## Results and discussions

### Optimized 3D structure of rat AFP

**Figure 3:**
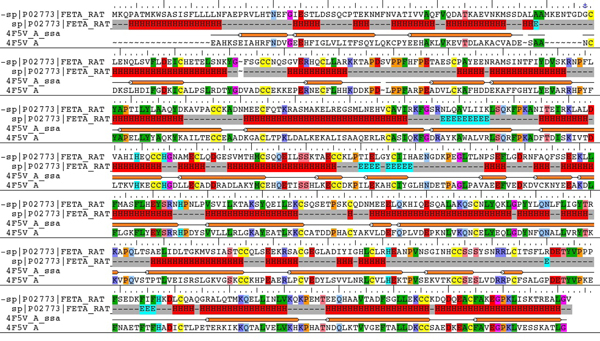
**Sequence alignment result between rat AFP and the template sequence of rabbit serum albumin**. The predicted secondary structure of rat AFP is also shown, where E represents for strand; H for helix; and - for loop. The identical residues are highlighted in colors according to the property of the corresponding query residue in the alignment to depict the location of charged, polar, and hydrophobic query residues.

With an identity of 34% and an alignment score of 1079, the sequence of rabbit serum albumin (Uniprot ID: G1U9S2) has the highest ranked homology in the BLAST search for rat AFP on proteins in PDB. Recent studies have demonstrated that 3D structures are similar if the sequence identity between two proteins is higher than 25% [30,31]. Therefore, the 3D structure of rat AFP built through homology modeling using the 3D structure of rabbit serum albumin as the template should be suitable for modeling rat protein binding data. The crystal structure of rabbit serum albumin was recently determined at a resolution of 2.27Å [21] and was deposited in PDB (PDB ID: 4V5F). The amino acid sequence of the rabbit serum albumin crystal structure is from Glu25 to Gly608 and covers the full length of rat AFP except for the 24 amino acids in the N terminus (Figure 3).

The initial alignment of rat AFP sequence with the template sequence was obtained using ClustalW. The alignment is consistent with experimental results [32] reporting that 15 disulphide bridges in the template structure are perfectly aligned to the rat AFP sequence (see Figure 3, where cystines are highlighted in yellow). The region of residues 70-110 in rat AFP could not be aligned with the template because rat AFP is larger. This region was manually adjusted according to the predicted secondary structures of rat AFP to avoid large gaps located inside the secondary structures of the template. The final alignment (32% identity, 51% positivity, and 4% gaps) used in the homology modeling and in the secondary structure prediction is shown in Figure 3. Besides the disulphide bridges, most of the secondary structures align well between the template and rat AFP. The initial homology models of rat AFP were built using Prime, with the model with the lowest energy used for further optimization.

**Figure 4:**
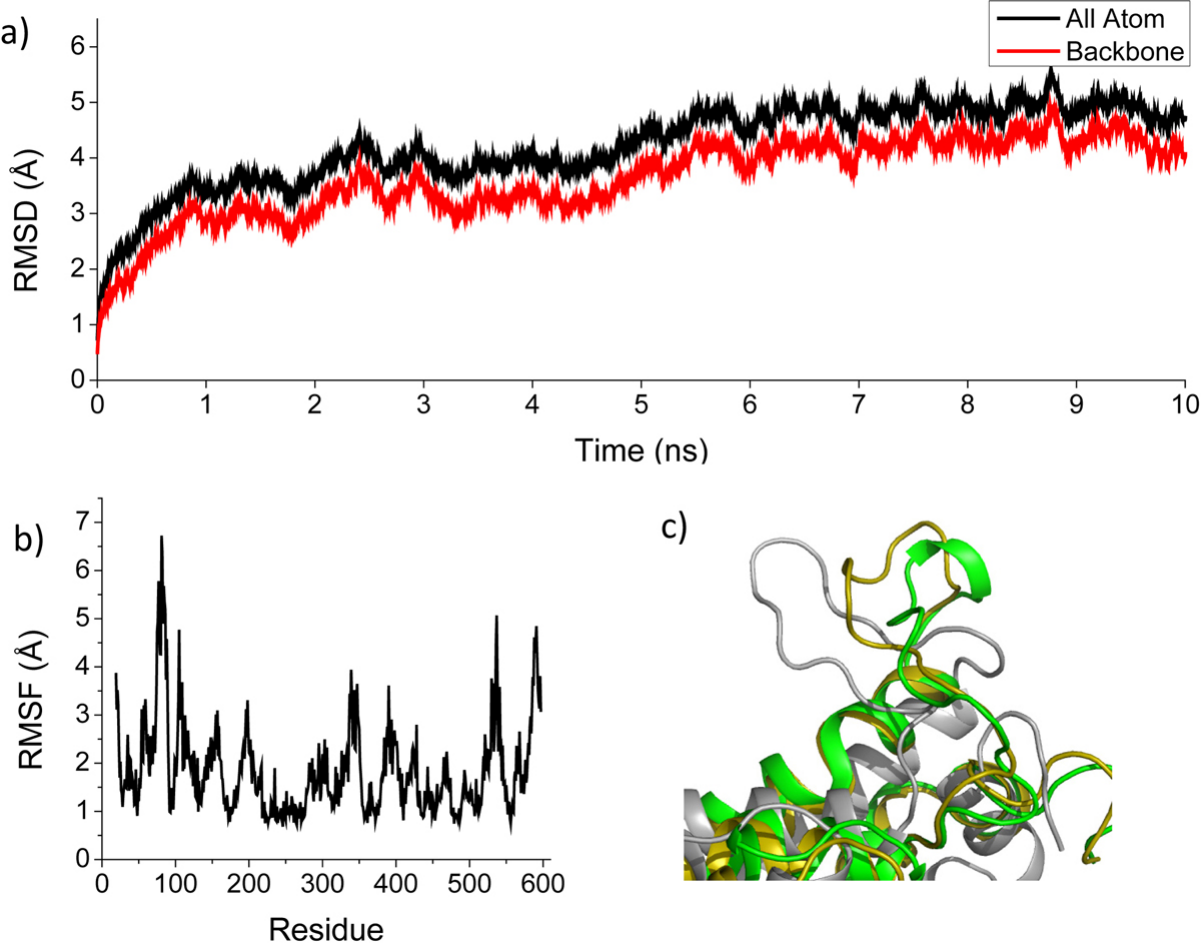
**MD profiles of the rat AFP tertiary structure optimization**. a) RMSD values (y-axis) along the time frame (x-axis) for atoms of protein backbone (black line) and ligand binding pocket (red line); b) RMSF values (y-axis) in MD simulation for individual along residues (x-axis); c) The loop from residues 70-90 folded to the helix after MD simulations (gray: initial models; yellow: after 5 ns MD simulation; green: after 10 ns MD simulation).

MD simulation has been commonly applied to refine homology models [33]. Herein, the initial 3D structure of rat AFP from homology modeling was optimized using MD simulation in solvent to mimic the real physiological environment. The stability of rat AFP structure during the MD simulation was measured by its deviation from the initial structure in terms of RMSD. The RMSD values of the rat AFP backbone atoms and all atoms in the entire MD simulation trajectory were as shown in Figure 4a. The 3D structures of rat AFP reached a stable state after 6 ns where the RMSD of the protein backbone atoms and of all atoms converged to 4 Å and 4.5Å, respectively. The root-mean-square fluctuations (RMSF) of all rat AFP structures generated during the MD simulation were calculated to characterize the mobility of individual residues as shown in Figure 4b. The side chain residues from 70 to 90 had high peaks in the RMSF plot (Figure 4b), indicating large fluctuations of those residues. Examining this region in the structures generated during the MD optimization revealed that the loop in the initial rat AFP structure folded into a small helix after the MD simulations (Figure 4c). The Ramachandran plot (Supplementary Figure S1 in additional file 1) of the optimized rat AFP structure showed that 99.3% of residues were placed in allowed regions, higher than the percentage of residues (98.6%) in allowed regions for the initial 3D rat AFP structure from homology modeling, indicating higher stability of the optimized rat AFP structure. The residues located in the favored zone were also increased from 91.1% to 92.5% after MD optimization. As a reference, the template crystal structure of rabbit serum albumin only had 92.2% and 99.0% residues in the favored and allowed zones, respectively. This indicates that the optimized rat AFP structure should be stable and reliable for use in elucidating of rat AFP-ligand binding modes through molecular docking and MD simulations.

### Binding modes generated by molecular docking

The two putative rat AFP binding sites proposed without the benefit of crystal structure data were speculative [16,17]. Herein, we used SiteMap [34] to search and analyze the potential ligand binding sites in rat AFP. To elucidate possible binding modes of different ligands, a large box was defined to cover all the potential binding sites generated by SiteMap. Therefore, the 13 ligands adopted the most favorable binding poses.

**Figure 5:**
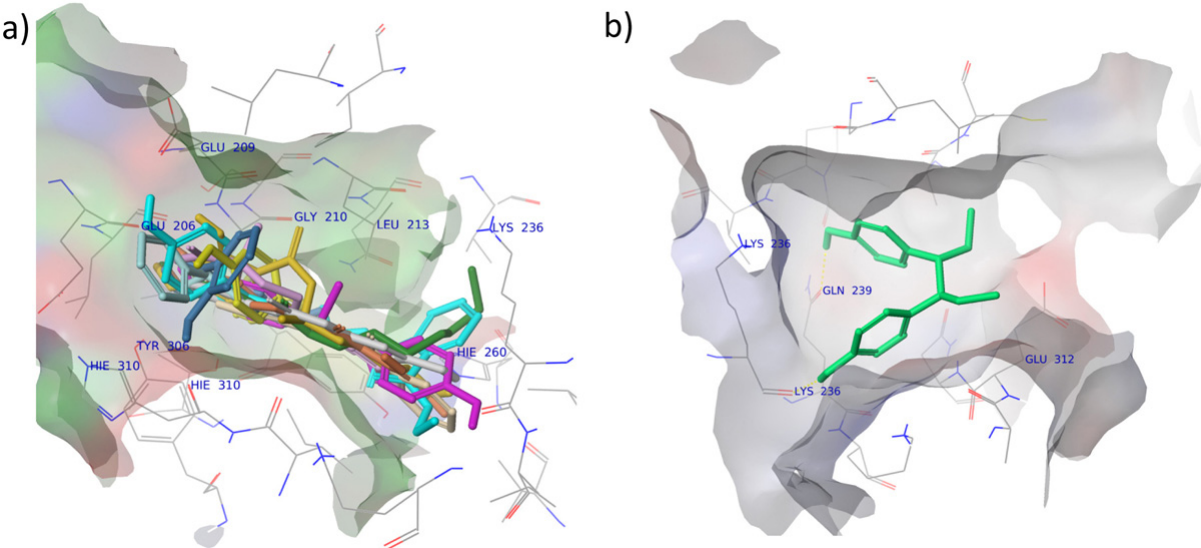
**Docking poses of the 13 rat AFP binders**. Binders are represented by constant colored sticks and protein are represented by lines and colored according atom types. The binding pocket surface is colored by electrostatic properties. a) Most ligands bind to the pocket comprising Glu206, Glu209, Gly210, Leu213, Lys236, His260, Try306, and His310; b) one ligand binds to the pocket composed of Leu236, Gln239 Tyr306, Ile309, His310, Glu312, Asn313, Leu359, Val361, Ala465 and Ile473.

Different binding poses of the 13 ligands were searched and ranked by docking analysis based on their docking scores. The pose with the lowest score, suggesting the most probable binding modes of a ligand, was selected for further analysis. The 13 rat AFP binders were successfully docked in a big pocket inside rat AFP. The preferable ligand binding poses for the 13 rat AFP binders are depicted in supplementary Figure S2 in additional. Twelve binders were docked in the site composed of residues Glu206, Glu209, Gly210, Leu213, Lys236, His260, Try306, and His310. The orientations of all 12 binders are similar: the hydrophobic part of the binders extends into the binding pocket, while the hydroxyl group-tethered part extends out to form hydrogen bonds with AFP (Figure 5a). Only one binder, DL-hexestrol, bound to the opposite site composed of Leu233, Gln239 and Glu312, in the same pocket. Two hydrogen bonds were formed between the two hydroxyl groups of DL-hexestrol and the ketone group of the Gln239 and Leu233 backbone. As shown in Figure 5b, DL-hexestrol folded in the binding site in the conformation that is not energy-favorable. The energy unfavorable binding mode might explain the weak binding affinity observed for DL-hexestrol.

The binding poses of the 13 rat AFP binders were assessed using the GScore scoring function comprised van der Waals energy, Coulomb energy, hydrophobic interactions, hydrogen bonding, polar interactions, and rotatable bonds penalty. The docking scores of the 13 rat AFP binders as well as their experimentally- determined binding affinities (IC_50_) are given in supplementary Figure S2. Quercetin had the lowest docking score. The docking results revealed that a hydrogen bonding network formed between the hydroxyl groups in the dihydroxybenzene part of quercetin and the residues Glu206, Tyr306 (supplementary Figure S2 in additional file 1). Such a hydrogen bonding network is likewise observed in the docking poses of coumestrol, α-zearalanol, diethylstilbestrol (DES), dioxybenzone, and heptyl p-hydroxybenzoate (supplementary Figure S2 in additional file 1). Estrone and 2,3,4,5-tetrachloro-4'-biphenylol were the two most potent rat AFP binders, and were also highest ranked in the docking analyses. Further examination of the bind poses indicated major contributions to their affinity are hydrophobic interactions.

### Binding modes refined by MD simulations

While molecular docking has been successfully used in predicting binding poses of ligands for many proteins, it has also failed in estimating of ligand binding affinity [35,36]. One of the major reasons for failure is treatment of proteins as "rigid" molecules in order to save computational time, and thus not allowing their conformations to adjust during docking. The rigidity assumption works well for proteins that lack flexibility. However, AFP is a very flexible protein, and AFP conformation change induced by some ligands has been reported [37,38]. MD simulation has been extensively applied to study conformation changes in protein-ligand interactions [39,40], protein dynamics [41,42], and protein folding [43,44]. Given AFP flexibility, MD simulations were deemed necessary to compare rat AFP conformation changes anticipated to differ across the 13 structurally diverse binders.

**Figure 6:**
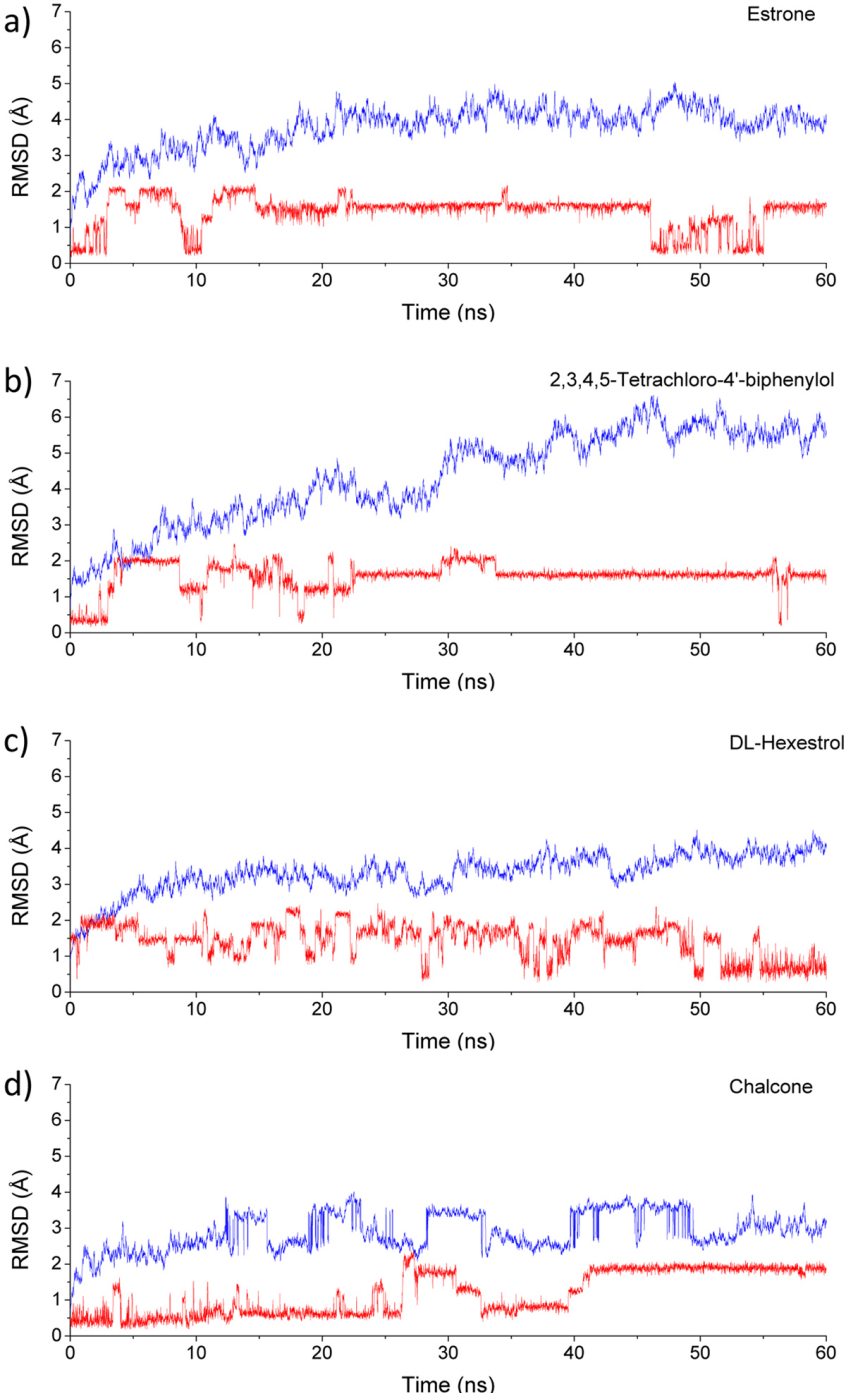
**RMSD in MD simulations**. RMSD of protein backbones are represented in blue lines, while RMSD of the ligands are depicted in red lines. a) the rat-AFP complex with estrone; b) the rat-AFP complex with 2,3,4,5-tetrachloro-4'-biphenylol; c) the rat-AFP complex with DL-hexestrol; d) the rat-AFP complex with chalcone.

In this study, MD simulations were carried out on the 13 rat AFP-ligand complex systemsto refine understanding of rat AFP binding modes obtained from molecular docking. The dynamic stabilities of the 13 complex systems were estimated using RMSD changes during the MD simulations as plotted in Figure 6 and supplementary Figure S3 in additional file 1. For the complexes of rat AFP bound with estrone, dihydorxymethoxychlorolefin, quercetin, coumestrol, heptyl p-hydroxybenzoate, DL-hexestrol and dioxybenzone, the protein was equilibrated with no obvious RMSD fluctuations observed after 20 ns. For the complexes of rat AFP bound with 2,3,4,5-tetrachloro-4'-biphenylol, DES and flavanone, RMSDs gradually increased in the first 50 ns and then converged in the time frame from 50 to 60 ns. The complex of rat AFP bound with α-zearalanol quickly equilibrated, though large RMSD fluctuations occurred during the last 5 ns, possibly due to the motions of the loop around Glu363 to Lys377. This loop is far from the ligand binding pockets and may thus minimally affect ligand binding. For the complexes of rat AFP bound with chalcone and diethyl phthalate (the two weakest rat AFP binders among the 13), large fluctuations were observed during the MD simulations (Figure 6d and supplementary Figure S3 in additional file 1). Interestingly, trajectory analysis revealed that diethyl phthalate exited the binding pocket after 10 ns, which is hardly surprising since weak binding affinity was measured for this binder in our previous study [4]. We also analyzed stability and conformation change with RMSD during the MD simulations of the 13 binders. Conformation changes were observed for all binders (see Figure 6 and supplementary Figure S3 in additional file 1). Moreover, binders with multiple rotatable bonds fluctuated more than rigid binders. For example, diethyl phthalate that contains two ester chains fluctuated considerably, while the rigid 2,3,4,5-tetrachloro-4'-biphenylol remained relatively stable during the MD simulations. Interestingly, binders with high binding affinity were stable even though they contained multiple rotatable bonds. For example, although dihydroxymethoxychloroolefin contains a rotatable n-heptane chain, its RMSD remained near 1 Å throughout the MD simulations. Estrone was stable during the MD simulation except for the time frame from 45 to 55 ns when it repositioned to another binding site in rat AFP, before shifting back after 55 ns. Because the binding pocket of rat AFP has more volume than some binders, such as coumestrol and flavanone, binders can reposition to different binding sites, exhibiting large fluctuations in RMSDs.

**Figure 7:**
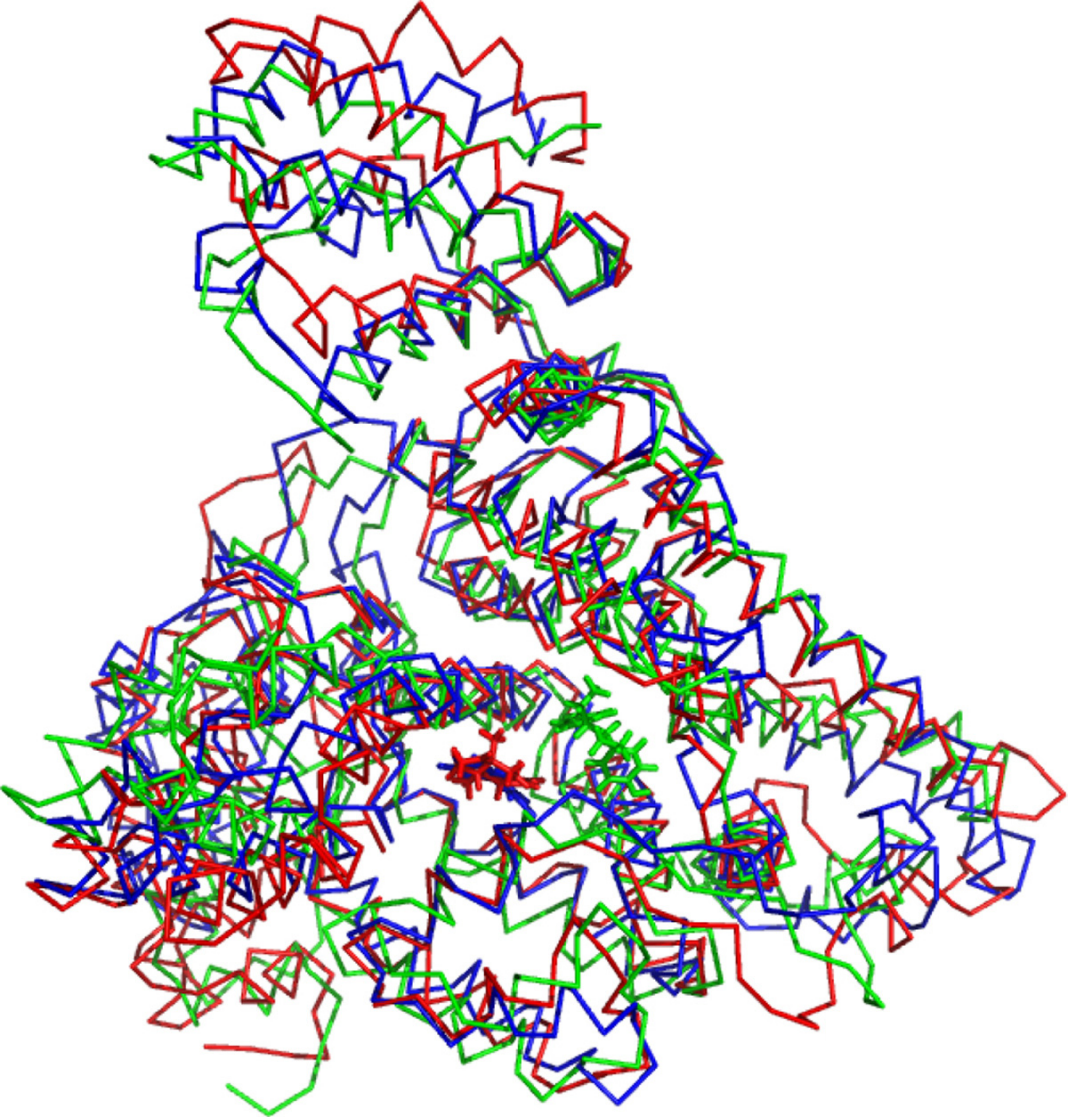
**The final snapshots (60 ns) of three ligand-protein complexes in the MD simulations (red: the rat-AFP complex with estrone; blue: 2,3,4,5-Tetrachloro-4'-biphenylol; green: the rat-AFP complex with chalcone)**. The proteins are represented using ribbon with constant color.

Ligand induced conformation changes were observed in our MD simulations. The superimposition of three bound complexes from the final snapshots of the MD simulations shown in Figure 7 (estrone: Red; 2,3,4,5-tetrachloro-4'-biphenylol: Blue; chalcone: Green) illustrates rat AFP conformation changes. Different ligands induced different conformation changes, though the MD simulations started from the same rat AFP conformation.

Diethyl phthalate was not able to stay in the binding pocket, exiting AFP 10 ns after the MD simulation. It was consequently excluded from the binding free energy calculation. The binding free energies were calculated for the rest of 12 binders using MM-GBSA methods based on the MD simulation trajectories from 50 to 60 ns. The overall binding free energy (ΔG_MM-GBSA_) and all energy terms from equations (2) through (5) from the MM-GBSA method are given in Table 1.

**Table 1:** Calculated Binding Free Energies in Comparison with Available Experimental Data (All in kcal/mol^a^)

Ligand	Δ*E*_electrostatic_	Δ*E*_vdw_	Δ*G*_GB_	Δ*G*_SA_	Δ*G*_elec_^b^	Δ*G*_np_^c^	ΔG_MM-GBSA_	ΔG_expt_^d^
estrone	-4.697	-33.641	-38.342	16.386	-43.039	-17.255	**-21.956**	-14.170
2,3,4,5-tetrachloro-4'-biphenylol	-10.820	-34.011	-44.831	13.439	-55.651	-20.571	**-31.391**	-13.499
dihydroxymethoxychlorolefin	-16.178	-35.206	-51.385	17.346	-67.563	-17.861	**-34.039**	-11.400
quercetin	-64.532	-28.019	-92.552	64.173	-157.084	36.154	**-28.378**	-11.299
diethylstilbestrol (DES)	-13.686	-38.031	-51.717	20.007	-65.404	-18.024	**-31.710**	-11.255
α-zearalanol	-44.887	-38.562	-83.449	48.873	-128.336	10.311	**-34.576**	-10.665
coumestrol	-19.018	-39.142	-58.160	23.885	-77.178	-15.257	**-34.275**	-10.472
heptyl p-hydroxybenzoate	-11.911	-30.747	-42.658	19.749	-54.569	-10.998	**-22.910**	-10.472
DL-hexestrol	-8.448	-29.507	-37.952	18.078	-46.400	-11.429	**-19.875**	-10.256
dioxybenzone	-5.791	-35.739	-41.530	17.046	-47.321	-18.694	**-24.485**	-10.033
flavanone	-4.047	-31.029	-35.076	13.354	-39.124	-17.675	**-21.722**	-9.511
chalcone	-15.359	-27.673	-43.033	18.745	-58.392	-8.929	**-24.288**	-8.766

^a ^Average over 1000 snapshots

^b ^Total electrostatic contribution: Δ*G*_elec_= Δ*E*_electrostatic_+ Δ*G*_GB_

^c ^Total nonpolar contribution: Δ*G*_np_= Δ*E*_vdw_+ Δ*G*_SA_

^d^Δ*G*_expt_=*RT***ln**IC_50_, T = 277K

To make direct comparisons between experimental binding affinities, (Δ*G*_exp_) was estimated from binding affinity data using ΔG_exp _≈ -*RT***ln**IC_50_[45] with results listed in Table 1. The reason we can make such approximation is that the dissociation constant K_d _is proportional to Z_reactants_/Z_products_, where Z is ensemble partition function. Consequently, the MM-GBSA binding free energies were much lower than the binding free energies estimated from experimental data. Analyzing the components in the MM-GBSA biding free energies indicated electrostatic energy to be the major contributor for binders with high polarity. For example, Δ*G*_elec _was -157 kcal/mol for quercetin that contains 5 hydroxyl groups, while Δ*G*_elec _was 128 kcal/mol for α-zearalanol that contains 3 hydroxyl groups. These two ligands have less unfavorable/positive nonpolar penalties Δ*G*_np _of 36 and 10 kcal/mol, respectively.

## Conclusions

AFP can change the availability of estrogenic chemicals to enter target cells. Knowledge of the binding affinity of an estrogenic chemical is important to estimate its potential endocrine activity. Such ER-mediated activity can be altered in rat through binding to AFP that sequesters estrogen in rat in circulating serum. The tertiary structures of AFP are crucial to understanding AFP-ligand interactions for evaluating chemical endocrine activity. Our results on binding interactions between chemicals and rat AFP would be helpful for further studies including evaluation of endocrine disruption potential of chemicals in the human environment, and designing more efficient drug products that target ER or compete with AFP sequestration of drugs.

## Disclaimer

The findings and conclusions in this article have not been formally disseminated by the US Food and Drug Administration (FDA) and should not be construed to represent the FDA determination or policy.

## Competing interests

The authors declare that they have no competing interests.

## Authors' contributions

JS and WZ performed all calculations and data analysis, and wrote the first draft of manuscript. HF and RK contributed to the data analysis, verified the calculations, and assisted with writing the manuscript. WT and HH developed the original idea and guided the data analysis and presentation of results. All authors read and approved the final manuscript.

## Supplementary Material

Additional file 1 **Supplementary Figures S1-S3**. Click here for file

## Declarations

Publication costs of this article were funded by the US government.

This article has been published as part of *BMC Bioinformatics *Volume 14 Supplement 14, 2013: Proceedings of the Tenth Annual MCBIOS Conference. Discovery in a sea of data. The full contents of the supplement are available online at http://www.biomedcentral.com/bmcbioinformatics/supplements/14/S14.
