# Supplementary material for: Homology modeling, molecular docking, and molecular dynamics simulations elucidated α-fetoprotein binding modes
Source: BMC Bioinformatics. 2013 Oct 9;14(Suppl 14):S6. doi: 10.1186/1471-2105-14-S14-S6 (PMC3851483; doi:10.1186/1471-2105-14-S14-S6)
Supplement: Additional file 1 — Supplementary Figures S1-S3. [file 1471-2105-14-S14-S6-S1.zip › 1471-2105-14-S13-S6-S1.pdf]

## Supplementary Figures

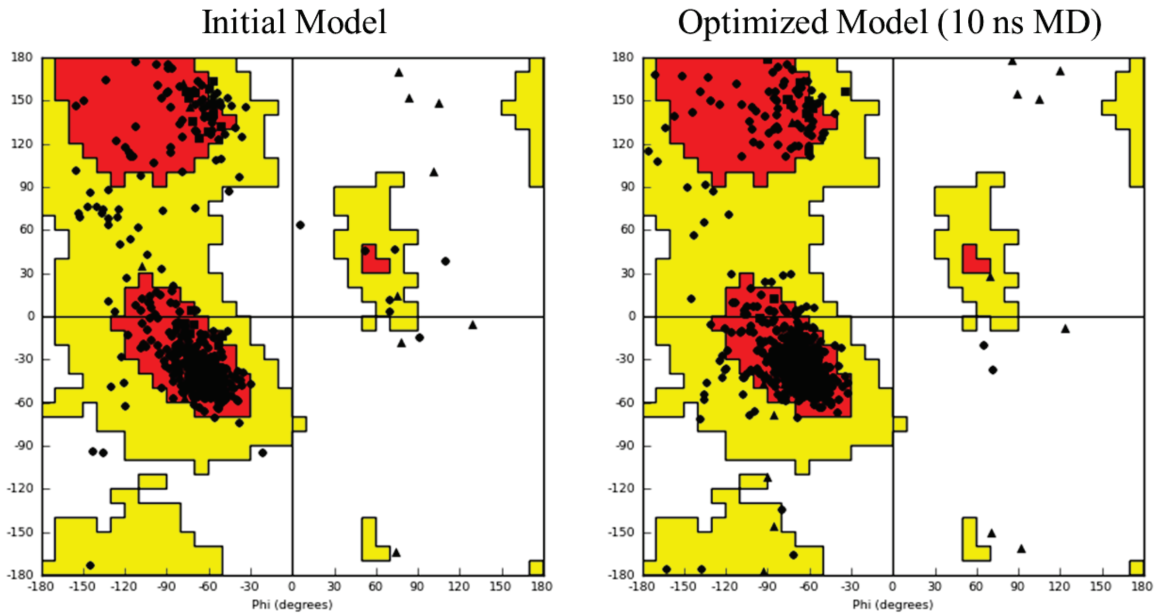


| | Initial | Optimized | *Template* |
|---|---|---|---|
| Favored Zone | 91.1% | 92.5% | *92.2%* |
| Allowed Zone | 98.6% | 99.3% | *99.0%* |
| Unfavored Zone | 1.4% | 0.7% | *1.0%* |

**Figure S1**TheRamachandran plots of the intial model and the optimized model. It provides the visualization of protein backbone through phi and psi angle. Each point in the plot is an amino acid residue. The triangle one represents glycine. 99.3% amino acids in the optimized model locate in the allowed zone, while only 98.6% in the allow zone for initial structure.

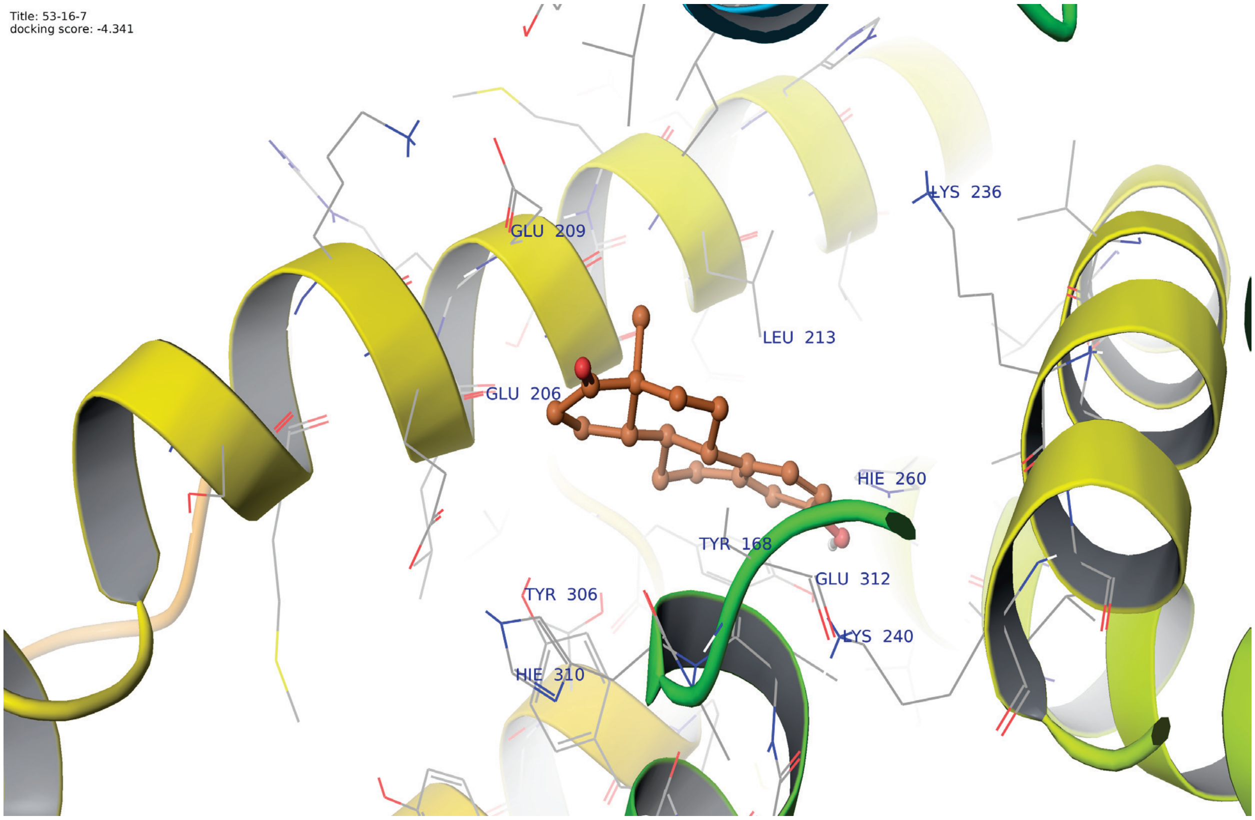

estrone

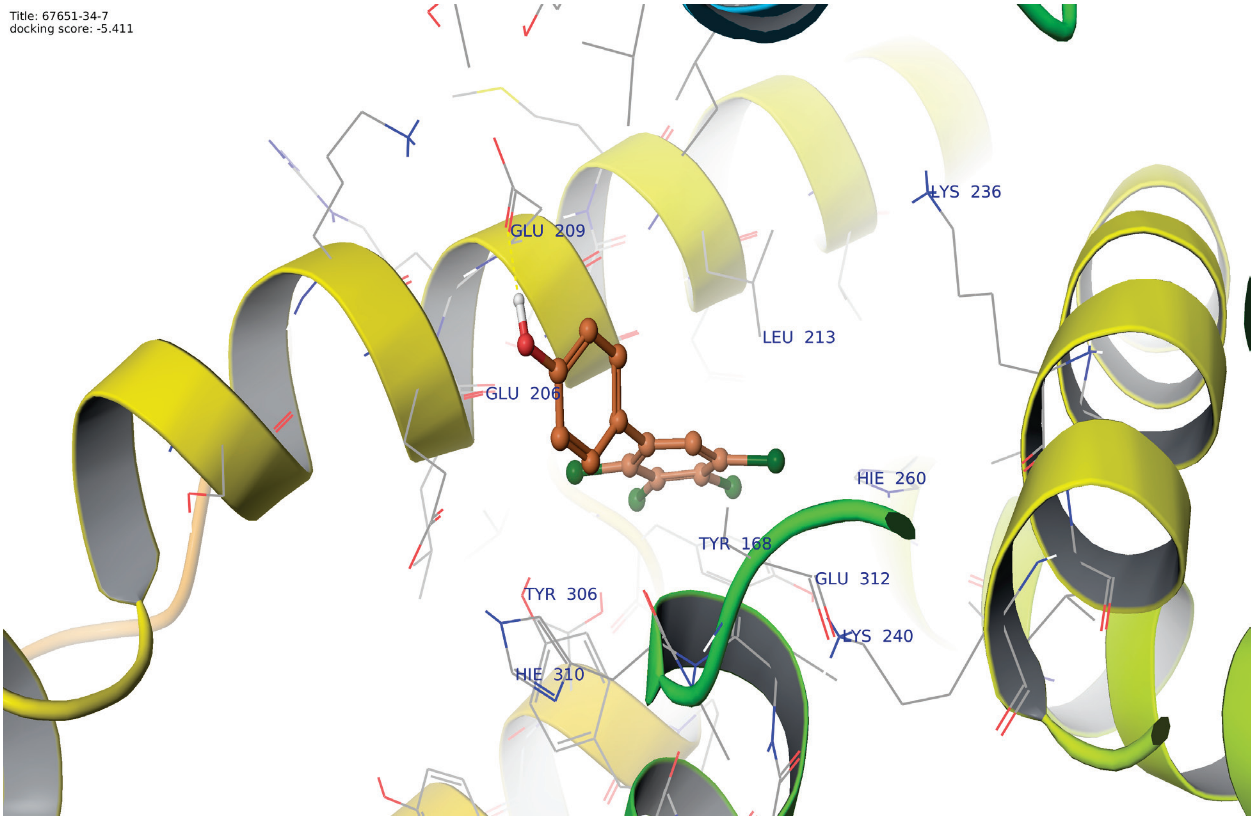

2,3,4,5-tetrachloro-4'-biphenylol

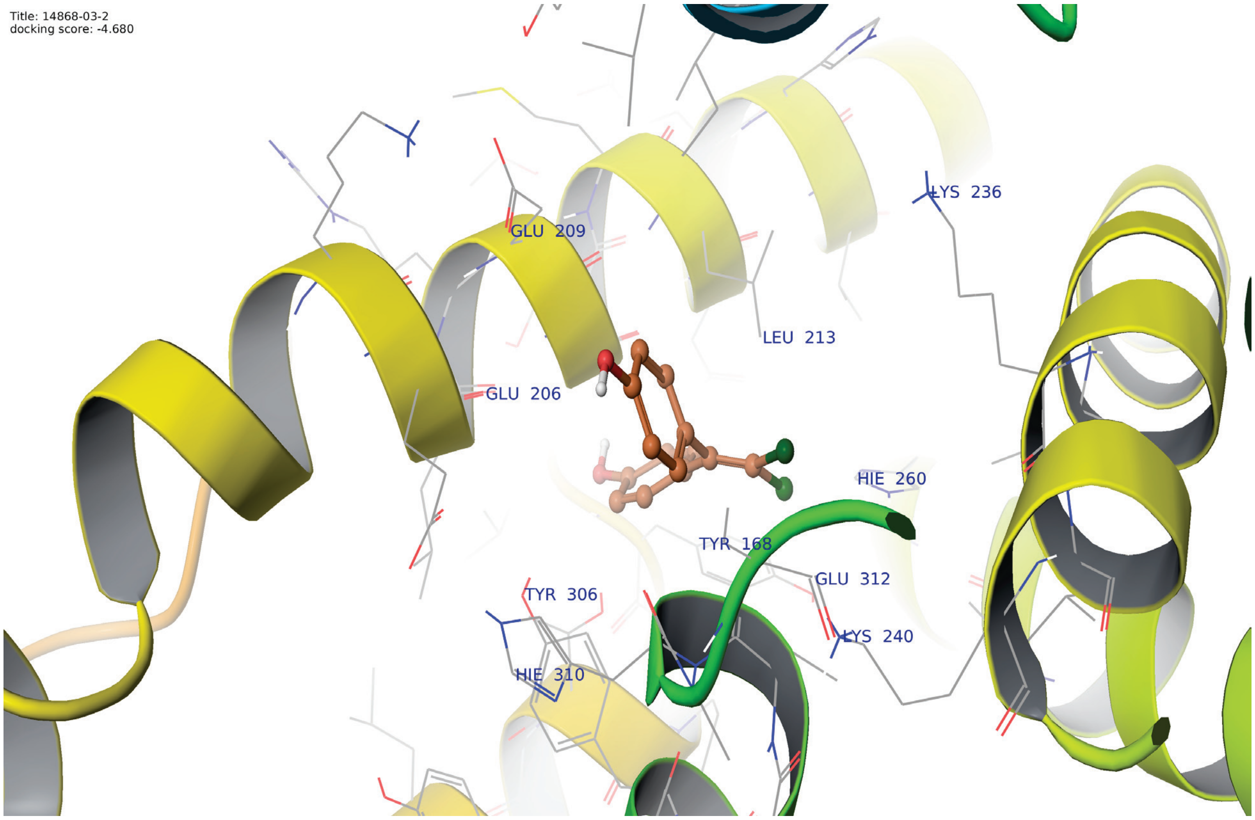


dihydroxymethoxychlorolefin

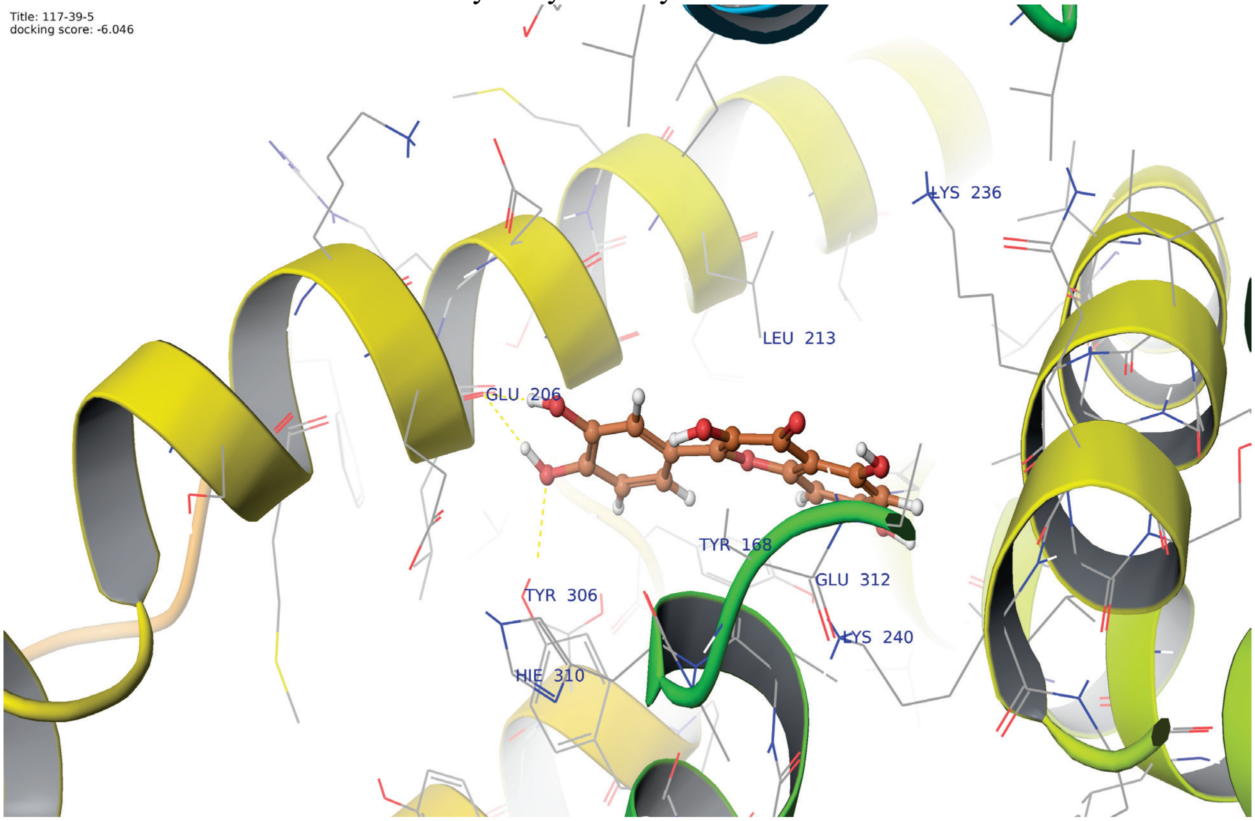


quercetin

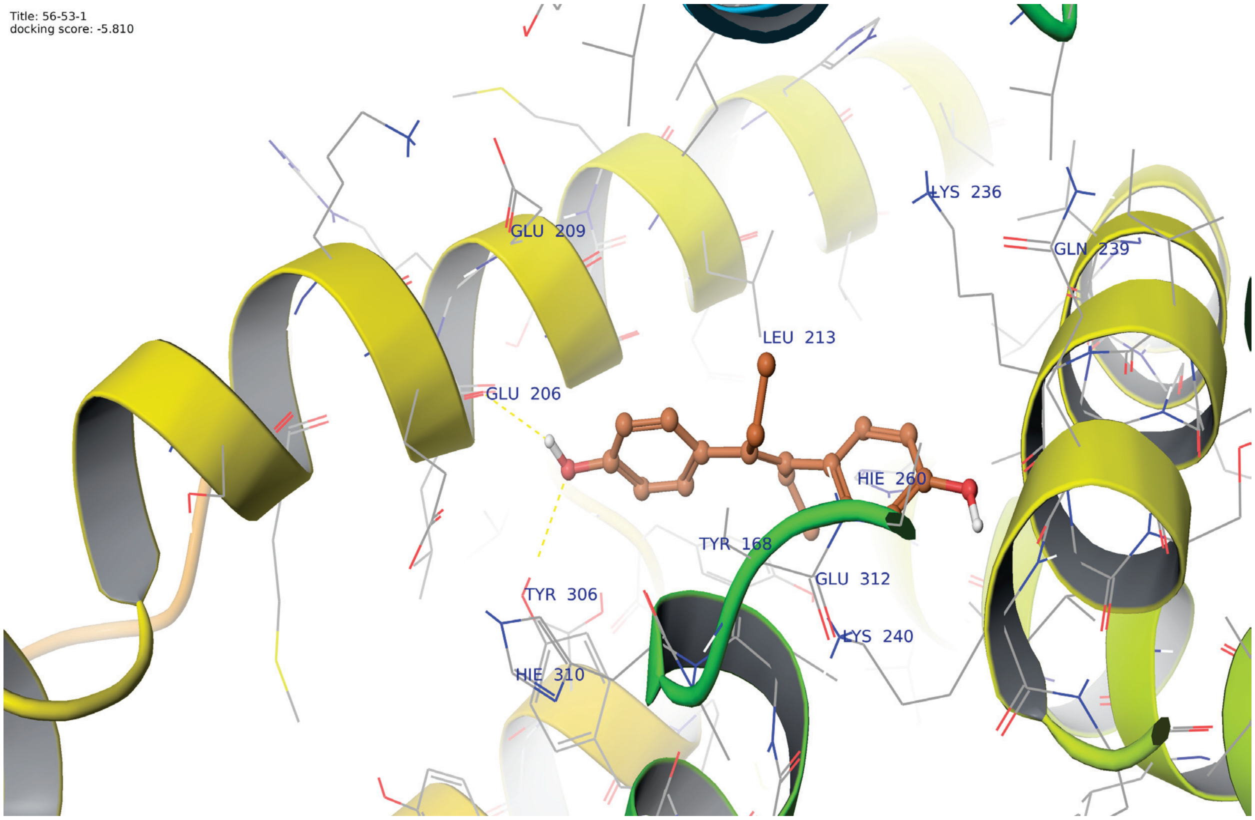


diethylstilbestrol (DES)

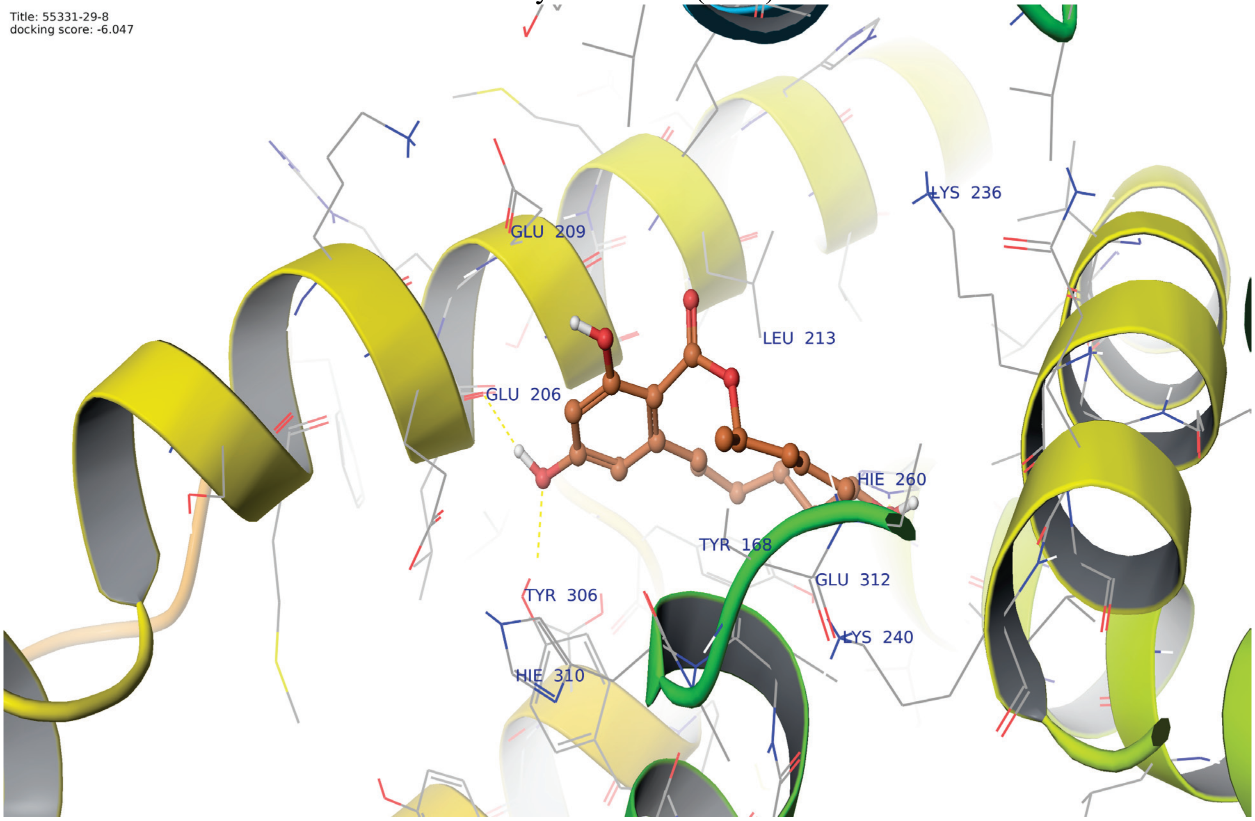


α-zearalanol

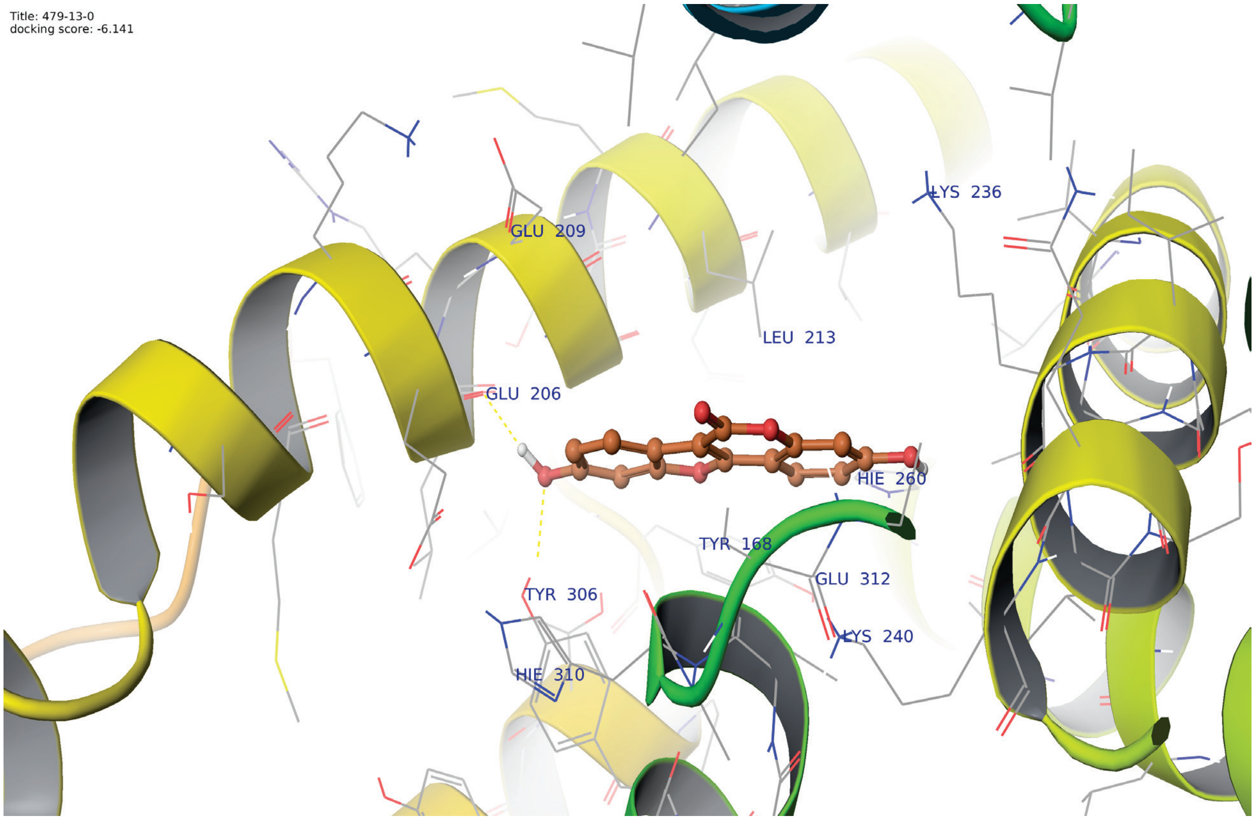

coumestrol

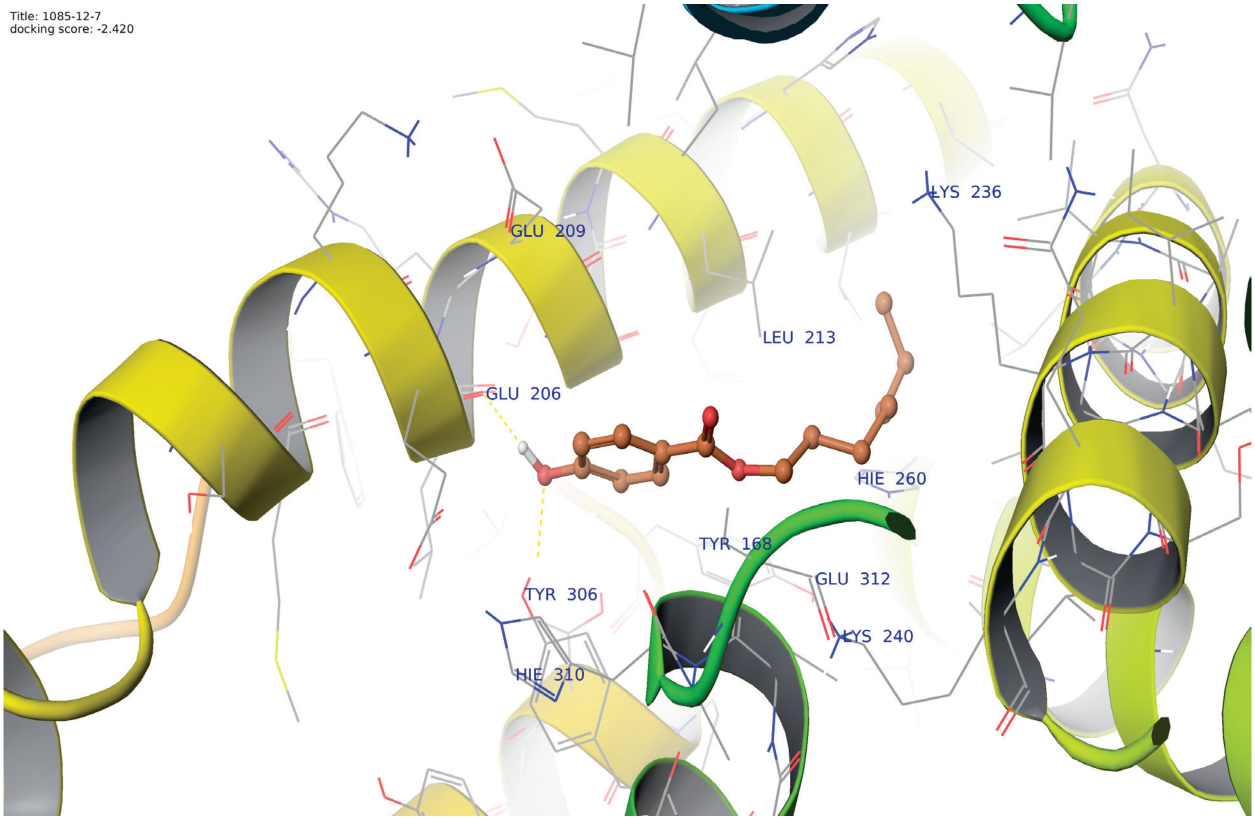

heptyl p-hydroxybenzoate

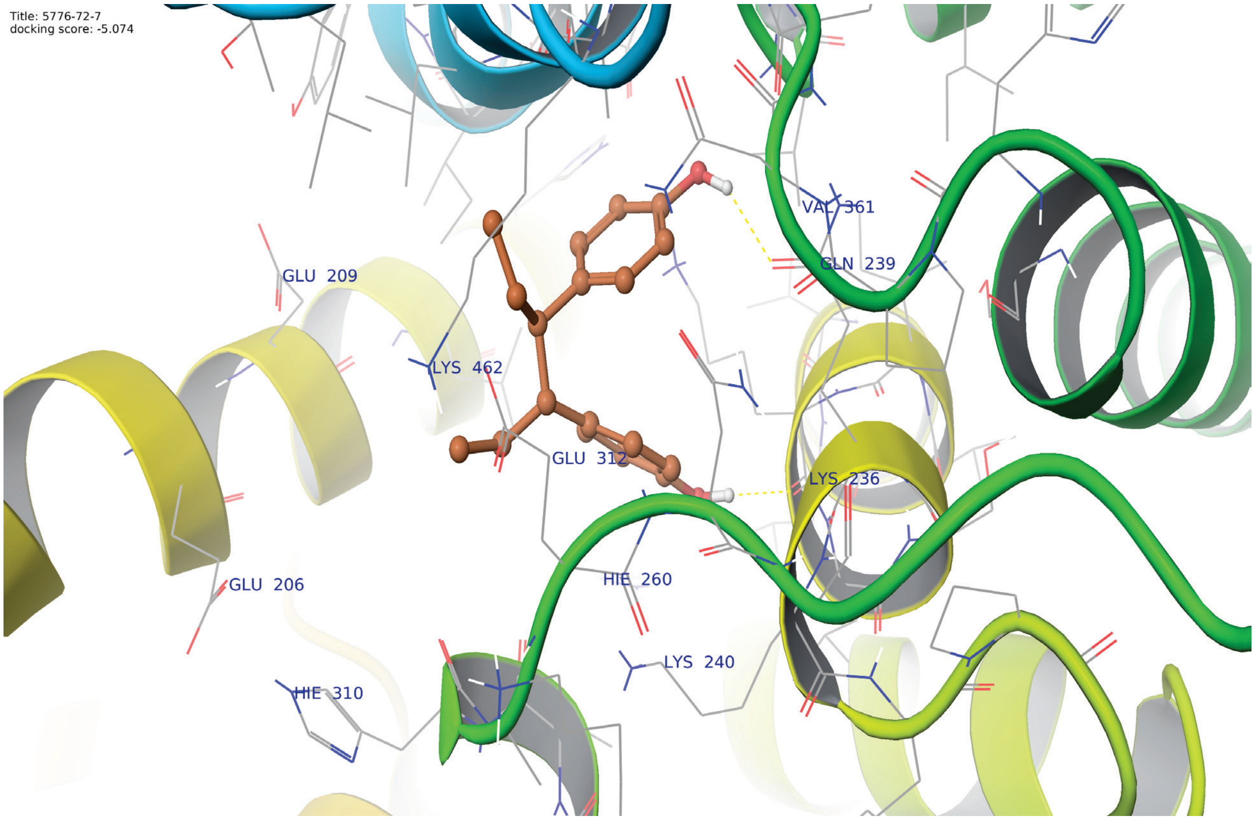


DL-Hexestrol

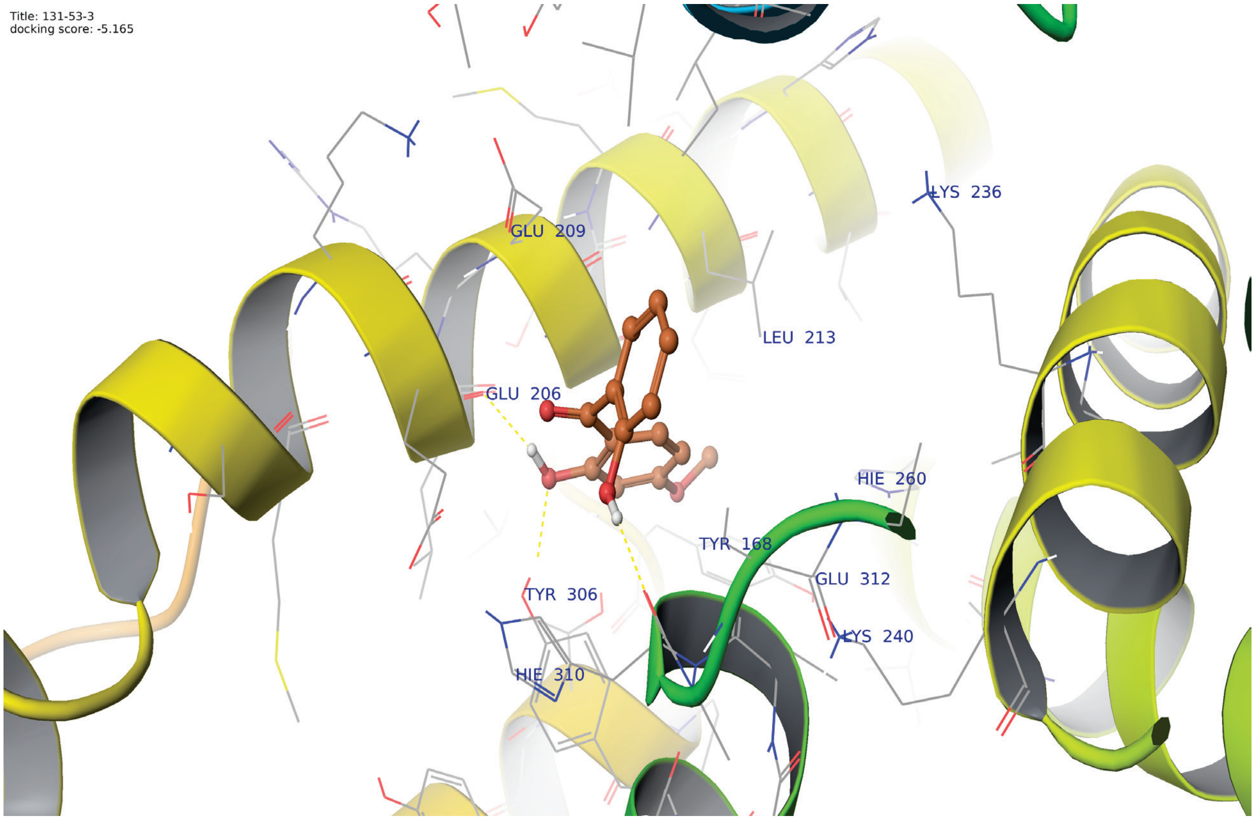


dioxybenzone

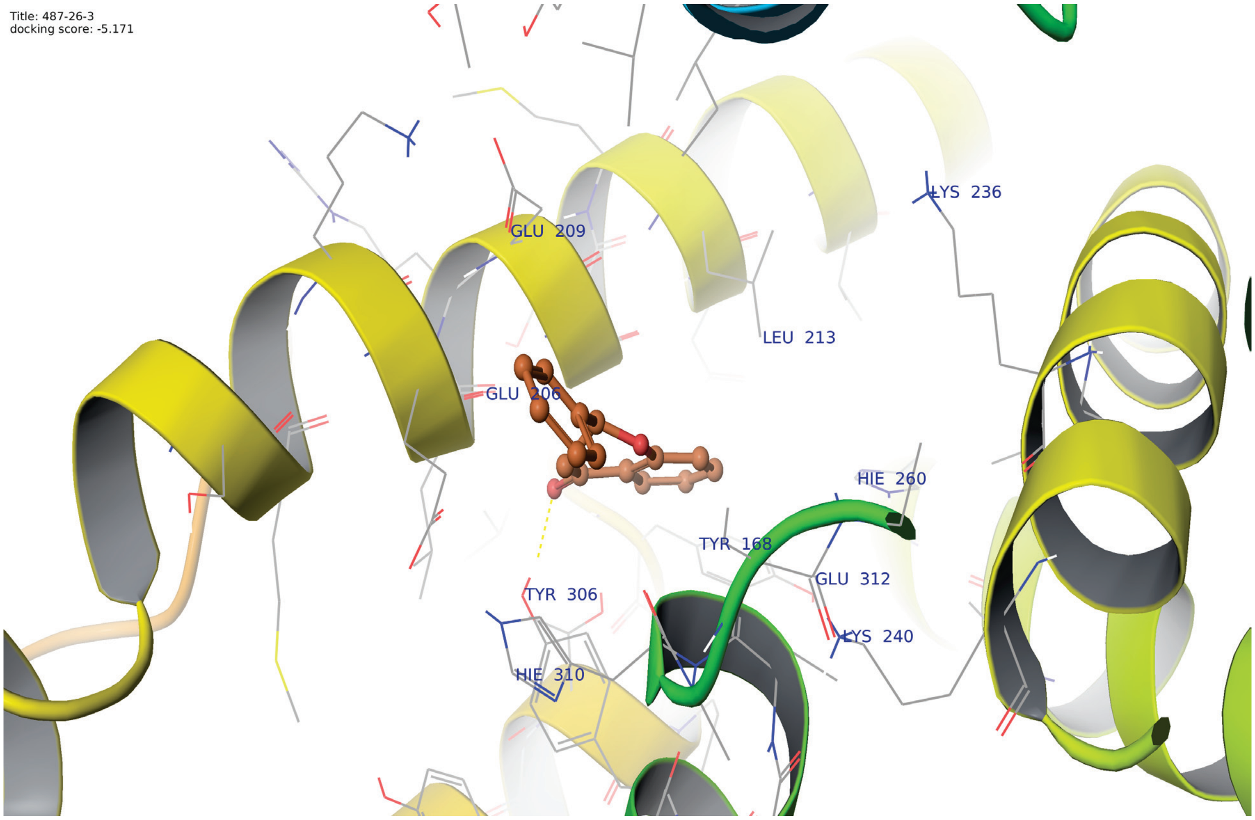


flavanone

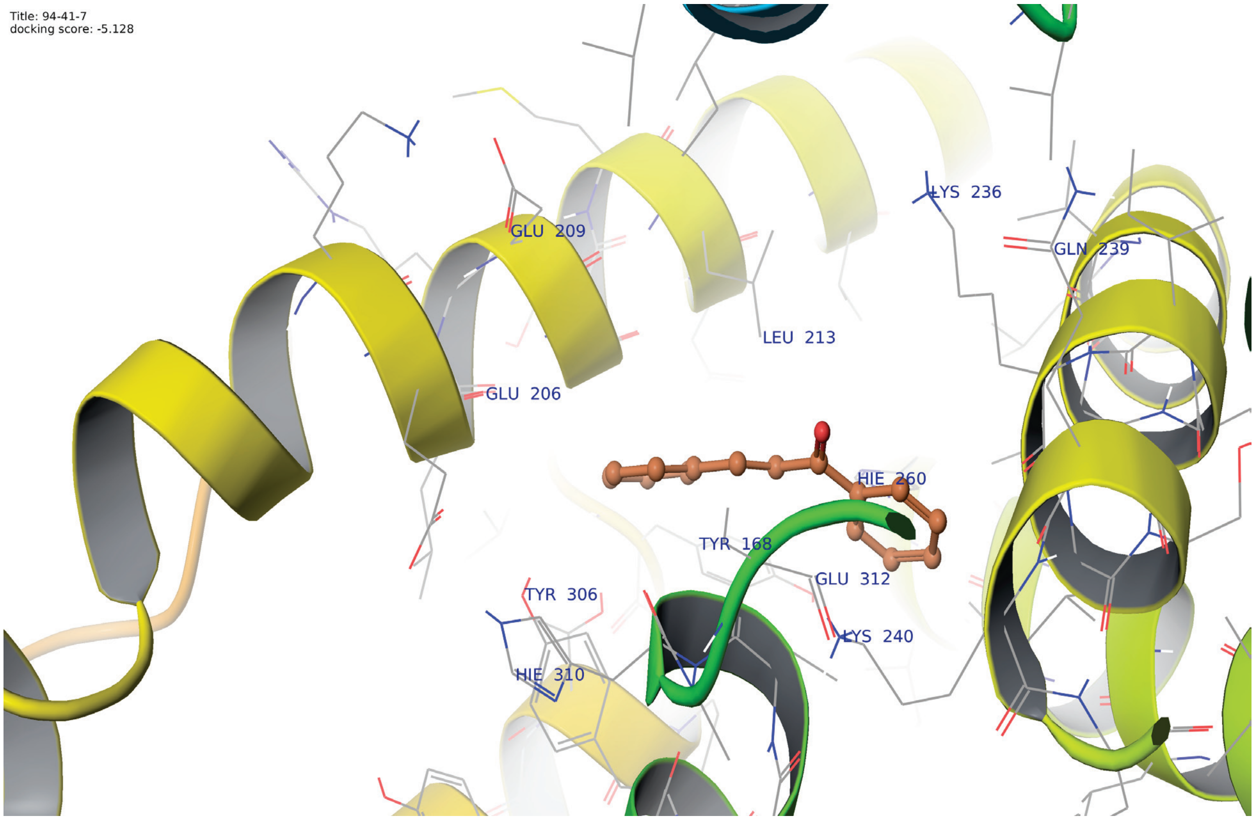


chalcone

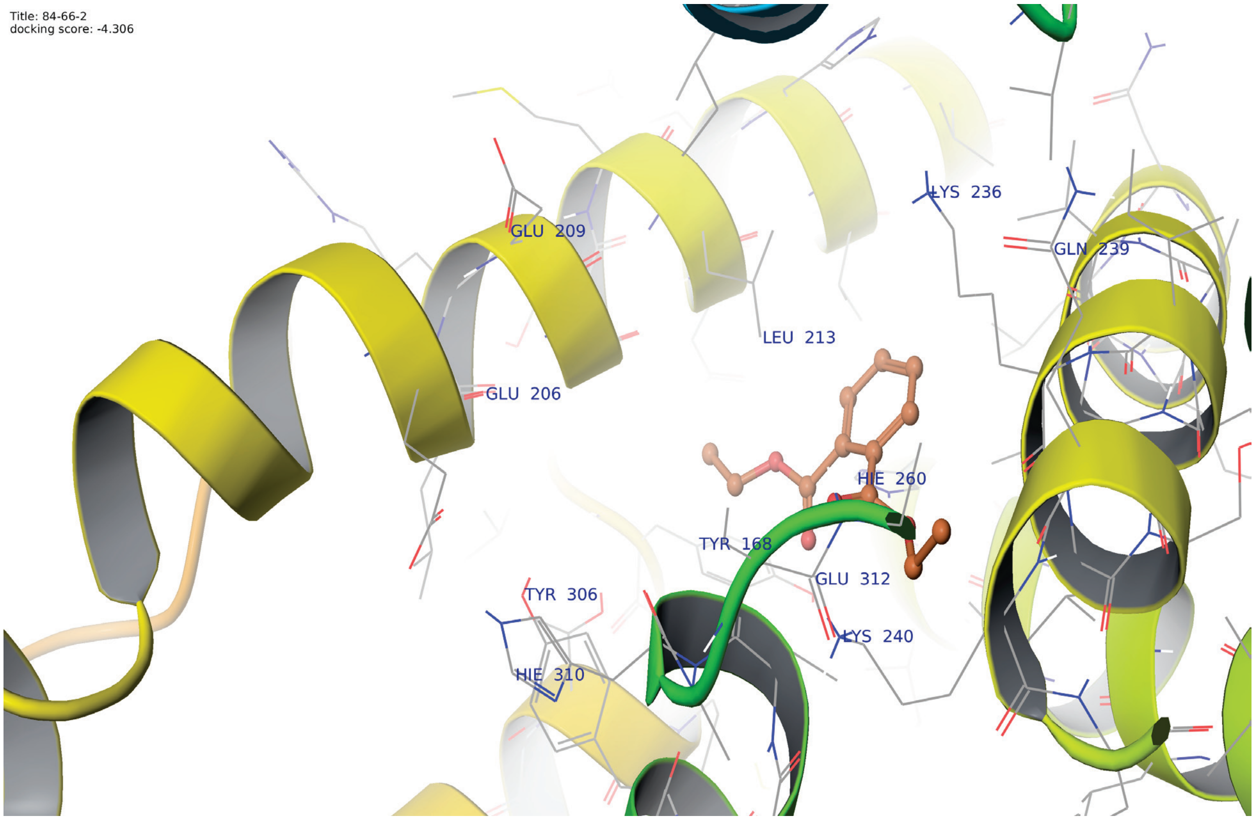


diethyl phthalate

**Figure S2** The initial binding poses of the 13 structural diverse rat AFP binders. Ligands are represented using ball and stick model. Hydrogen bonds are representing using yellow dash lines.

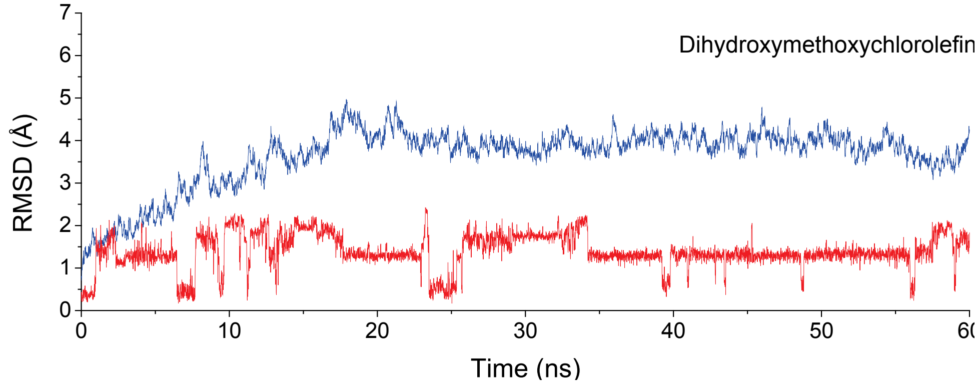
Dihydroxymethoxychlorolefin
RMSD (Å)
Time (ns)
0 1 2 3 4 5 6 7
0 10 20 30 40 50 60

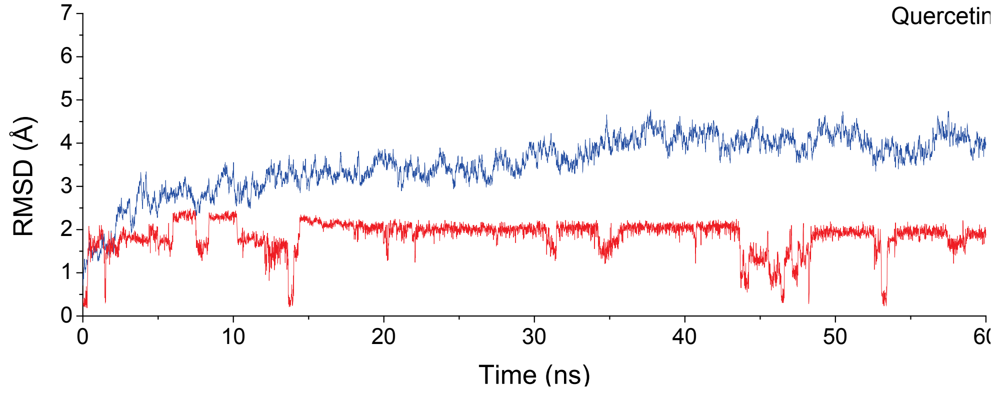
Quercetin
RMSD (Å)
Time (ns)
0 1 2 3 4 5 6 7
0 10 20 30 40 50 60

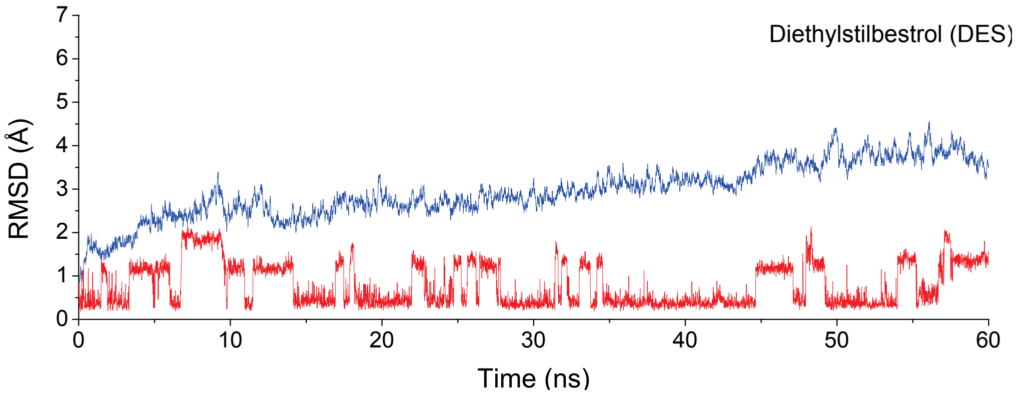
Diethylstilbestrol (DES)
RMSD (Å)
Time (ns)
0 1 2 3 4 5 6 7
0 10 20 30 40 50 60

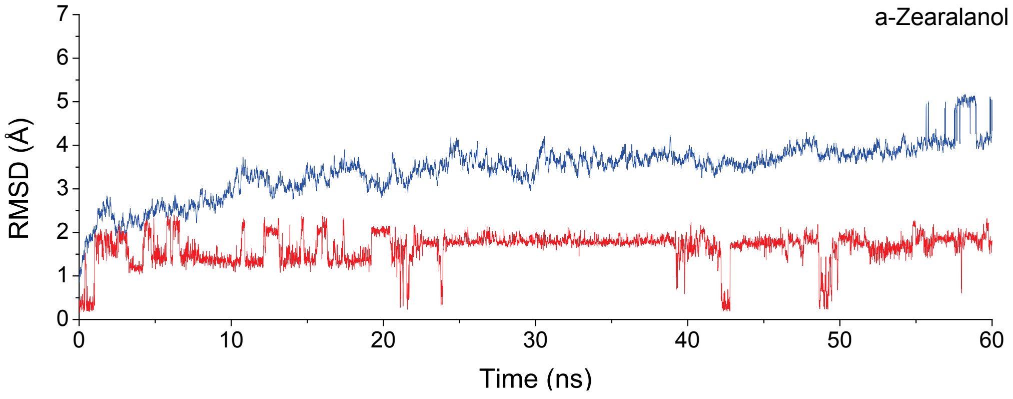
a-Zearalanol
RMSD (Å)
Time (ns)
0 1 2 3 4 5 6 7
0 10 20 30 40 50 60

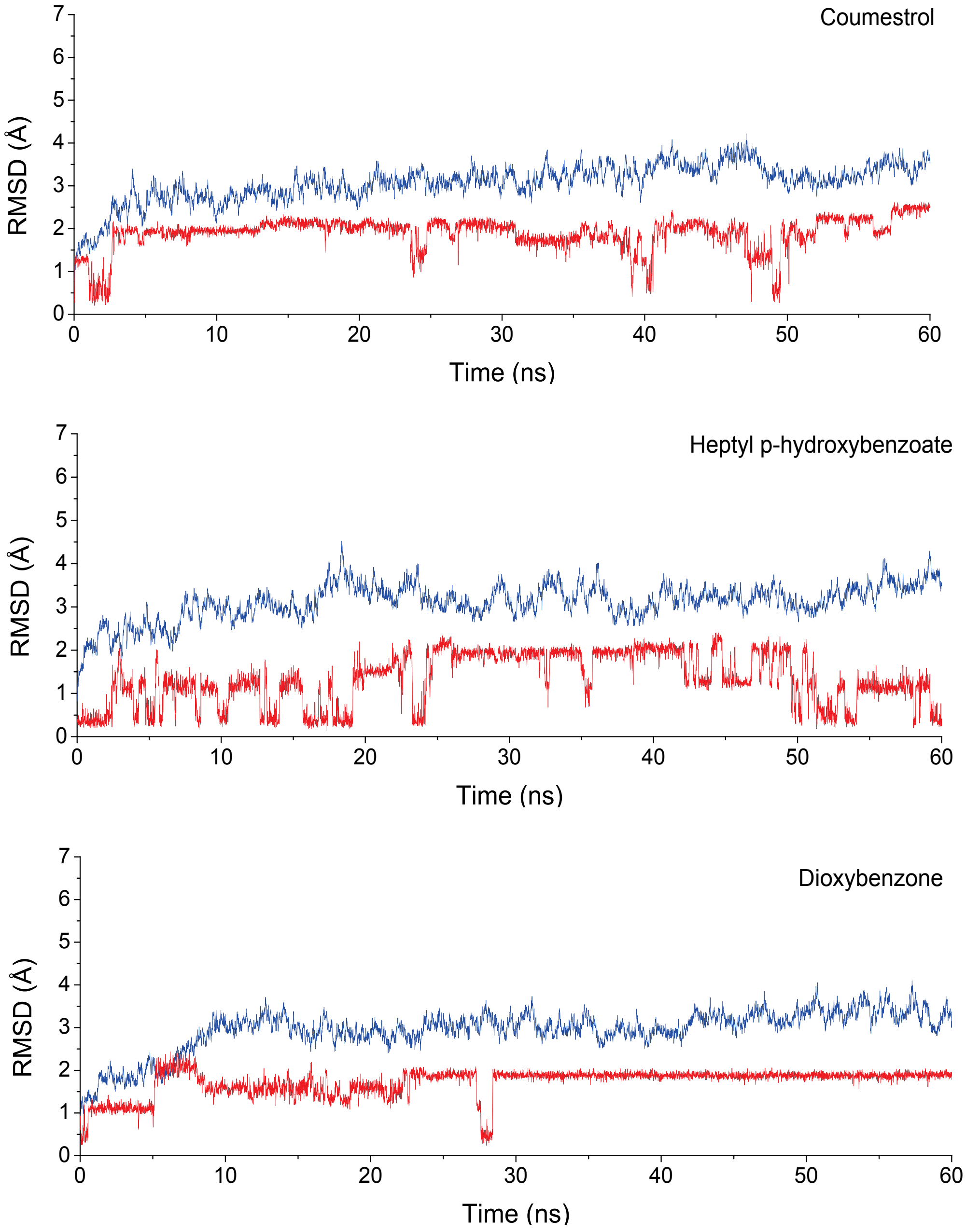
Coumestrol
RMSD (Å)
Time (ns)
Heptyl p-hydroxybenzoate
RMSD (Å)
Time (ns)
Dioxybenzone
RMSD (Å)
Time (ns)

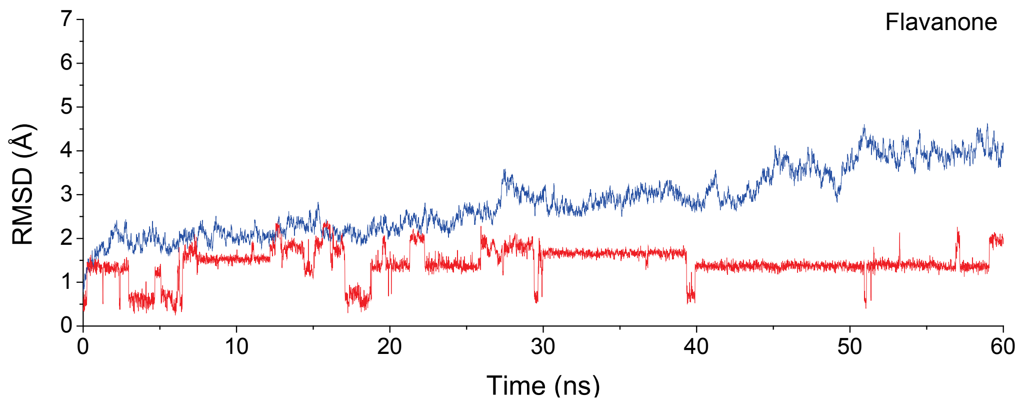


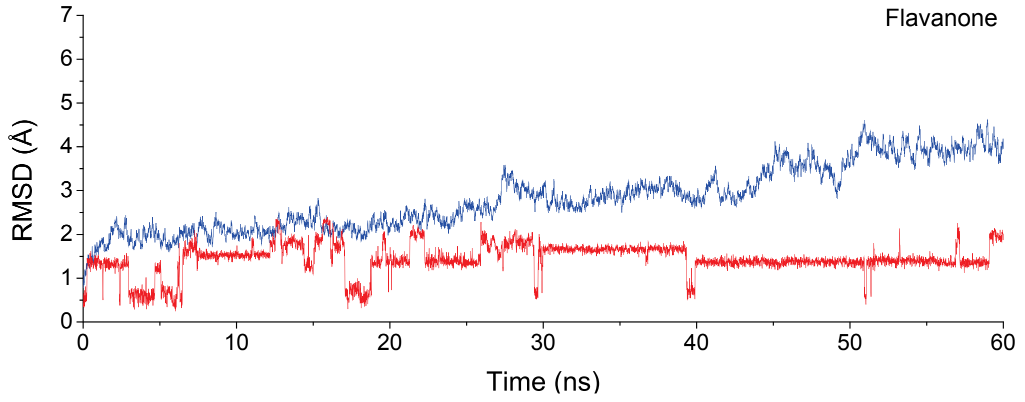


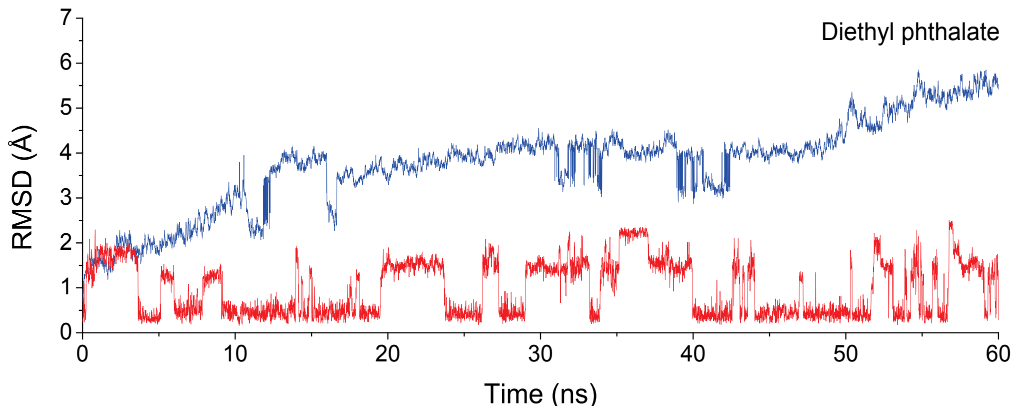


**Figure S3** The RMSD versus MD simulation time for the 9 ligand-protein complex systems. Protein backbones were represented using blue lines, and the ligands were represented using red lines.
